# Appropriateness and Safety of Direct Access Endoscopy in Hospitalized Patients

**DOI:** 10.7759/cureus.11453

**Published:** 2020-11-12

**Authors:** Felicia R D'Souza, Aymen Almuhaidb, Dayna Early, Osama Altayar, Mark Thoelke

**Affiliations:** 1 Hospital Medicine, University of Chicago Pritzker School of Medicine, Chicago, USA; 2 Gastroenterology and Hepatology, Washington University School of Medicine, St. Louis, USA; 3 Hospital Medicine, Washington University School of Medicine, St. Louis, USA

**Keywords:** diagnostic efficacy, gastrointestinal endoscopy, direct access endoscopy, hospitalists, inpatients, diagnosis, gastroenterology, referral and consultation

## Abstract

Background and objective

Direct access endoscopy (DAE) allows hospitalists to refer patients for endoscopy without a gastroenterologist (GI) evaluation, potentially decreasing wait time and facilitating earlier discharge from the hospital. This study aimed to evaluate the efficacy and safety of DAE for average-risk endoscopic procedures.

Methods

A retrospective chart review was performed by comparing patients who underwent a DAE with patients who underwent an endoscopy ordered by GI physicians at a tertiary care hospital. The procedure indications were obtained from the endoscopy reports and hospitalist progress notes. Appropriateness of each procedure was determined based on the guidelines from the American Society for Gastrointestinal Endoscopy (ASGE). Findings, procedure-related complications, and clinical significance were recorded.

Results

A total of 110 patients were included in this study; 40 were DAE and 70 were ordered by GI. The mean age of the patients was 55.5 years with 69 males and 41 females. In the DAE group, there were 31 esophagogastroduodenoscopies (EGD) and nine colonoscopies performed, while in the GI group, there were 58 EGDs, 11 colonoscopies, and one push enteroscopy. All procedures fulfilled ASGE criteria; 20/40 DAE and 53/70 GI-ordered procedures had clinically significant findings. There was one complication in each group.

Conclusion

DAE allows a hospitalist to order an endoscopy without consultation with a GI physician. This study showed that all DAE procedures had met ASGE criteria for appropriateness, with 50% having clinically significant findings and no difference in adverse events. These results suggest that DAE is safe and effective in evaluating hospitalized patients for average-risk endoscopy.

## Introduction

Direct access endoscopy (DAE) is a diagnostic procedure that allows hospital medicine physicians (hospitalists) to refer patients to inpatient endoscopy without prior evaluation by a gastroenterologist (GI). DAE was initially introduced to allow primary care physicians to quickly refer patients for endoscopic evaluation when they presented with gastrointestinal symptoms, thereby reducing cost and wait time for patients [1]. Although DAE is widely available in outpatient settings, this study is the first to explore the effectiveness of DAE in an inpatient setting in the United States.

The main goals of DAE in an inpatient setting include decreasing wait time, improving patient turnover, and facilitating earlier discharge from the hospital. At most academic centers, a GI consult is initially seen by the fellow and then staffed by an attending later in the day. Endoscopies are then performed the following day. DAE allows for endoscopies to be performed on the same day. At our center, inpatient DAE has been available since 2000. Hospitalists refer stable patients for DAE when there are no contraindications for the procedure or other complex issues that would have to be cleared by a GI. In this study, we aim to evaluate the safety and efficacy of inpatient DAE and evaluate its adherence to the American Society for Gastrointestinal Endoscopy (ASGE) guidelines [2].

## Materials and methods

We conducted a retrospective manual chart review to compare patients who underwent a DAE requested by hospitalists with patients who underwent an endoscopy ordered by GI consult physicians at a tertiary medical center from August 2017 through December 2018.

DAE was first introduced in our tertiary hospital in 2000 when primary care groups were given the opportunity to order endoscopy without prior GI consultation in order to improve efficiency. At our medical center, hospitalists contact the nurse in charge of endoscopy to schedule the inpatient procedure. Average-risk procedures are performed without prior evaluation by a GI unless there are clear contraindications for the procedure or other complex issues (e.g., morbid obesity or substance abuse). Some relative contraindications for the procedure that have been considered include severe neutropenia, coagulopathy, severe thrombocytopenia, and increased risk of perforation including connective tissue disorder [3]. Requesting physicians have to secure appropriate intravenous access and ensure the patient’s ability to provide informed consent prior to the procedure [4]. If patients are unable to provide consent, contact information for the power of attorney (POA) is obtained to allow the endoscopist to gain consent for the procedure. Hospitalists are advised to pursue pre-procedural testing based on medical history, physical examination, and patients’ risk factors based on guidelines [5]. The target laboratory parameters prior to each endoscopic procedure are as follows: hemoglobin: >7 grams per deciliter (g/dL); platelets: >50,000 per microliter; international normalized ratio (INR): <2; sodium level: >130 millimoles/liter (mmol/L); and potassium level: 3-5 mmol/L.

In addition to patient demographics, the type of procedure and indications for each procedure were obtained from endoscopy reports and hospitalist progress notes prior to each procedure. For the DAE group, all procedural indications were obtained from the hospitalists’ progress notes or from the history and physical admission notes from the day prior to the procedure. All indications for the GI group were obtained from the endoscopy reports or GI consult notes. The appropriateness of each procedure was determined based on guidelines from the ASGE [2]. Findings from each endoscopy were recorded and clinical significance was determined. We defined clinically significant endoscopic findings based on the indications for the procedure and the presence of findings that were relevant to the indication. For instance, esophageal stenosis findings on the esophagogastroduodenoscopy (EGD) was a clinically significant finding for a patient who presented with dysphagia/odynophagia (Table 3). We also evaluated the safety of the procedure by recording the findings and major complication(s) that occurred during and after the procedure from the endoscopy reports and daily progress notes.

Scheduled outpatient procedures performed by the inpatient endoscopists were excluded from the study. Statistical analysis was conducted using R software [6]. Continuous variables were summarized as medians with interquartile intervals, and binary variables were summarized as frequencies. The outcomes of significant endoscopic findings and incidences of adverse events were compared using chi-squared and Fisher’s exact tests. The study was approved by the Human Research Protection Office at our institution.

## Results

A total of 110 patients were included in this study, of which 40 were DAE ordered by hospitalists, and 70 were ordered by GI physicians. The demographics and procedure details are presented in Table 1.

**Table 1:** Demographic data and procedure details GI: gastroenterology; EGD: esophagogastroduodenoscopy

Variables		Hospitalist	GI
Number of procedures		40	70
Type of procedure	EGD	31	58
	Colonoscopy	9	11
	Enteroscopy	0	1
Gender	Male	21	48
	Female	19	22
Age (years)	Mean age	57.65	54.50
	Median age	56	57

The most common indication for procedures in the DAE group was abdominal pain/dyspepsia followed by anemia, while that in the GI group was melena followed by hematemesis and hematochezia (Table 2); 29 cases had two indications for the procedure listed on the chart. Both indications were reviewed and both met ASGE criteria and were clinically appropriate.

**Table 2:** Indications for endoscopy DAE: direct access endoscopy; GI: gastroenterology; IBD: inflammatory bowel disease

Indication		DAE (%)	GI-ordered (%)
No indication provided		0	0
Gastrointestinal bleeding evaluation	Anemia	25%	2.30%
	Hematemesis	3.85%	13.79%
	Hematochezia	1.92%	12.64%
	Melena	5.77%	25.29%
	Evaluate fecal-occult blood	0	1.15%
Stomach and intestinal issues	Abdominal pain/dyspepsia	25%	5.75%
	Persistent nausea/vomiting	3.85%	5.75%
	Weight loss	7.69%	5.75%
	Dysphagia/odynophagia	19.23%	10.34%
	Evaluation of colitis	0	1.15%
	IBD evaluation	0	3.45%
Surveillance/evaluation	Follow-up for varices	0	6.90%
	Surveillance of polys or malignancy	3.85%	3.45%
Other	Chest pain	0	0
	Foreign body removal	0	0
	Other	3.85% (2 abnormal imagings)	2.30% (1 diarrhea, 1 abnormal imaging)

All of the procedures in both groups met the ASGE criteria, with 73 procedures having clinically significant findings. Of these 73 procedures, 20 were DAE (of which 50% had clinically significant findings) and 53 were ordered by GI physicians (of which 76% had clinically significant findings) (p-value: 0.006). Esophagitis was the most common finding, followed by gastric erosions/ulcers, normal findings, and esophageal varices (Table 3). Findings were deemed to be clinically significant if they directly contributed to the indication (see Table 3 for clinically significant vs. non-significant findings). Hemorrhoids were an incidental finding in the majority of the cases except for three cases where patients had large bleeding hemorrhoids causing hematochezia.

**Table 3:** Findings noted on endoscopy based on indications H. pylori: Helicobacter pylori

Procedure indication		Findings considered clinically significant	Findings considered clinically non-significant
Gastrointestinal bleeding evaluation	Anemia	Gastric ulcer (3)	Normal (4)
			Non-erosive gastritis, negative H. pylori (2)
			Non-erosive duodenitis (1)
			Diverticulosis (1)
			Colonic polyps (2)
			Mild colonic inflammation (1)
			Small non-bleeding hemorrhoids (1)
	Hematemesis	Esophageal varices (2)	Esophageal erosions (1)
		Esophagitis (5)	Non-erosive gastritis, negative H. pylori (1)
		Gastric ulcer (3)	
		Duodenal ulcer (2)	Gastric erosions (2)
		Duodenal angiodysplasia (1)	Non-erosive duodenitis (1)
			H. pylori (1)
			Hiatal hernia (1)
			Colonic polyps (2)
	Hematochezia	Esophageal varices (2)	Normal (1)
		Esophageal ulcer (2)	Grade A esophagitis (1)
		Duodenal ulcer (1)	Non-erosive gastritis (1)
		Large bleeding hemorrhoids (3)	Non-erosive duodenitis (1)
			Hiatal hernia (1)
		Colon angiodysplasia (1)	Mild colonic inflammation (2)
			Colonic polyps (4)
	Melena	Esophageal varices (3)	Normal (2)
		Grade C and D esophagitis (5)	Grade A esophagitis (2)
			Non-erosive gastritis, H. pylori-negative (3)
		Gastric ulcer (5)	
		Duodenal angiodysplasia (1)	Gastric erosion (2)
			Gastric polyps (1)
			Duodenal erosions (1)
			Non-erosive duodenitis (1)
			Hiatal hernia (2)
Stomach and intestinal issues	Abdominal pain/dyspepsia	Grade A–D esophagitis (5)	Normal (2)
		H. pylori-induced gastritis (1)	Hiatal hernia (1)
			Mild non-erosive gastritis, negative H. pylori (6)
		Gastric ulcer (1)	Non-erosive duodenitis (1)
			Hiatal hernia (1)
			Mild colonic inflammation (1)
			Colonic polyp (1)
			Small non-bleeding hemorrhoids (1)
	Nausea/vomiting	Candida esophagitis (1)	Normal (1)
		Gastric ulcer (1)	Gastric erosions (1)
		Esophageal ulcer (1)	Non-erosive gastritis, negative H. pylori (3)
			Hiatal hernia (1)
	Weight loss	Esophageal ulcer (2)	Normal (2)
			Grade A esophagitis (2)
			Duodenal erosions (1)
			Non-erosive gastritis, negative H. pylori (1)
			Non-bleeding hemorrhoids (2)
	Dysphagia/odynophagia	Candida esophagitis (1)	Normal (5)
		Grade C and D esophagitis (6)	Gastric polyps (1)
			Hiatal hernia (1)
		Severe esophageal stenosis (2)	Non-erosive gastritis (2)
			Gastric ulcer (2)
		Malignant-appearing distal esophageal stenosis (1)	Non-erosive duodenitis (1)
		Benign, esophageal stricture (1)	
		Esophageal mass (2)	
		Esophageal ulcer (1)	
Surveillance/evaluation	Follow-up for varices	Esophageal varices (5)	Grade A esophagitis (2)
			Gastric polyp (1)
	Surveillance of polyps or malignancy	Colonic polyps (3)	Hiatal hernia (1)

There were two patients with complications: one patient had an apneic episode during the procedure (DAE patient); the other patient had Forrest criteria 2A gastric ulcer of a major vessel that started oozing after the first endoscopic hemoclip was placed (GI patient) [7]. Hemostasis was achieved in this patient after a second endoscopic hemoclip was placed and there were no further complications.

## Discussion

Endoscopy is a valuable diagnostic and therapeutic tool that is used to diagnose and treat gastrointestinal disorders. DAE makes it easier for hospitalists to refer patients for endoscopy without prior consultation with a GI physician. Since the first use of open-access-endoscopy in 1974 [8], there has been a significant increase in its use in the outpatient setting. DAE in the outpatient setting has been widely believed to be more convenient for patients while reducing cost and waiting time for patients [1,9]. To our knowledge, this is the first study of DAE use in an inpatient setting in the United States.

Our study showed that all of the procedures requested by hospitalists had met ASGE criteria for appropriateness, with 50% having clinically significant findings. There was no difference in adverse events between the groups. A study that evaluated inpatient endoscopy requested by inpatient non-GI physicians in Dublin, Ireland from November 2012 to January 2013 reported that 27/30 (90.0%) of the requested studies were appropriate. A referral was considered appropriate based on ASGE guidelines as well as criteria pre-determined by GI consultants in their department [10]. Findings from our study are consistent with the Dublin study, and both studies seem to indicate that hospitalists have a good awareness of endoscopy referral indications.

Our study has some limitations. The nursing staff at our endoscopy lab follow a protocol to confirm appropriate vital signs and acceptable basic labs prior to arranging transport for the patient to the endoscopy lab. If there are concerns that the case might not be appropriate, the nurses consult the GI physicians about canceling or rescheduling the procedure. We were unable to identify such cases as they were not available in the endoscopy unit records. Another possible limitation is that the procedure indication obtained from the endoscopy report could be considered as biased, as the endoscopist might choose an indication that would match ASGE guidelines to get appropriate billing. To adjust for this, we evaluated the progress notes from hospitalists on the day prior to the endoscopy and found that the hospitalist diagnosis had met ASGE criteria for an appropriate indication in all cases.

This article was previously presented as a meeting abstract at the 2019 American College of Gastroenterology Annual Meeting on October 29, 2019 [Abstract: P2180 - Appropriateness and Safety of Direct Access Endoscopy (DAE) in Hospitalized Patients. American College of Gastroenterology Annual Meeting; October 29, 2019].

## Conclusions

DAE allows a physician to send a patient for an endoscopy without prior consultation with a GI physician. Our results suggest that DAE is safe and effective in evaluating hospitalized patients for average-risk endoscopy.
